# Neuronal degeneration in autonomic nervous system of *Dystonia musculorum *mice

**DOI:** 10.1186/1423-0127-18-9

**Published:** 2011-01-28

**Authors:** Kuang-Wen Tseng, Mei-Lin Peng, Yang-Cheng Wen, Kang-Jen Liu, Chung-Liang Chien

**Affiliations:** 1 School of Optometry, College of Medical Sciences and Technology, Chung Shan Medical University, Taichung, Taiwan; 2 Department of Ophthalmology, Chung Shan Medical University Hospital, Taichung, Taiwan; 3 Department of Anatomy and Cell Biology, College of Medicine, National Taiwan University, Taipei, Taiwan

## Abstract

**Background:**

*Dystonia musculorum *(*dt*) is an autosomal recessive hereditary neuropathy with a characteristic uncoordinated movement and is caused by a defect in the *bullous pemphigoid antigen 1 *(*BPAG1*) gene. The neural isoform of *BPAG1 *is expressed in various neurons, including those in the central and peripheral nerve systems of mice. However, most previous studies on neuronal degeneration in *BPAG1*-deficient mice focused on peripheral sensory neurons and only limited investigation of the autonomic system has been conducted.

**Methods:**

In this study, patterns of nerve innervation in cutaneous and iridial tissues were examined using general neuronal marker protein gene product 9.5 via immunohistochemistry. To perform quantitative analysis of the autonomic neuronal number, neurons within the lumbar sympathetic and parasympathetic ciliary ganglia were calculated. In addition, autonomic neurons were cultured from embryonic *dt/dt *mutants to elucidate degenerative patterns *in vitro*. Distribution patterns of neuronal intermediate filaments in cultured autonomic neurons were thoroughly studied under immunocytochemistry and conventional electron microscopy.

**Results:**

Our immunohistochemistry results indicate that peripheral sensory nerves and autonomic innervation of sweat glands and irises dominated degeneration in *dt/dt *mice. Quantitative results confirmed that the number of neurons was significantly decreased in the lumbar sympathetic ganglia as well as in the parasympathetic ciliary ganglia of *dt/dt *mice compared with those of wild-type mice. We also observed that the neuronal intermediate filaments were aggregated abnormally in cultured autonomic neurons from *dt/dt *embryos.

**Conclusions:**

These results suggest that a deficiency in the cytoskeletal linker BPAG1 is responsible for dominant sensory nerve degeneration and severe autonomic degeneration in *dt/dt *mice. Additionally, abnormally aggregated neuronal intermediate filaments may participate in neuronal death of cultured autonomic neurons from *dt/dt *mutants.

## Background

*Dystonia musculorum (dt) *is an autosomal recessive hereditary neuropathy in mice caused by the ablative *bullous pemphigoid antigen 1 *(*BPAG1*) gene [1]. The human homologue of the mouse sequence from the *dt *locus is on chromosome 6p12 [2]. Heterozygous *dt *mice appear normal phenotypically, but homozygous *dt *mice develop dystonia. Young *dt/dt *mutants are typically smaller than their normal littermates, and at approximately two weeks after birth, they exhibit abnormal postures and progressive loss of movement coordination. Hyperflexion and pronation of foot paws are other symptoms [3,4]. Previous studies have demonstrated substantial degenerative alterations involving the peripheral and central sensory pathways, and spinal motor neurons are slightly affected [5]. This pathology appears primarily related to abnormal axonal accumulations of cytoskeleton in *dt/dt *mice [5-8].

The cytoskeletal interacting protein, BPAG1, appears in several isoforms in different tissues [9]. The neural isoform of *BPAG1 *mRNA, *BPAG1n*, has been detected in a variety of neuronal systems during normal growth, such as in neurons within dorsal root ganglia, trigeminal ganglia, sympathetic ganglia, enteric nerve system, and spinal ventral horns [5]. *BPAG1n *is generally expressed in neurons in numerous regions in wild-type mice, but not all neurons deficient in *BPAG1 *cause serious degeneration in *dt/dt *mice [5]. Most previous studies on neuronal degeneration in *dt/dt *mice focused on the sensory nerve system [3,5], whereas the autonomic nervous system was seldom addressed. In our previous study of spinal motor neurons in *dt/dt *mice, no significant neuronal loss was observed in the spinal motor neurons [8]. However, the lifespan of these homozygous mutants is limited to three to four months. In human peripheral neuropathy, some evidences have indicated that sensory and autonomic neurons undergo degeneration together [10,11]. Autonomic neuronal degeneration and sensory deficiency are assumed to play key roles in the early mortality of *dt/dt *mice.

Investigations have revealed that the cytoskeletal interacting protein, BPAG1n, interacts with microtubules, microfilaments and neuronal intermediate filaments (IFs) and plays an important role in maintaining cytoarchitectural integrity [9,12-14]. Pathological changes in *dt/dt *axonal degeneration have been found together with aggregation of IFs [5,7]. Moreover, studies in transgenic mice and in transfected stable cell lines that overexpress neuronal IF have demonstrated abnormal IF accumulation in degenerating neurons [15,16]. These results may also be significant to neuronal diseases, in which IF protein aggregation plays an important role in neuronal degeneration. Abnormal IF protein aggregations in the cytoplasm are critical because the hyperphosphorylation of cytoplasmic IFs may trigger the neuronal death [17-19]. In clinical neuropathy, neurodegenerative disorders are morphologically represented by progressive neuronal degeneration and associated typical cytoskeletal change [20,21]. In addition, degenerative neurons with neuronal cytoplasmic inclusions have been observed in neuronal intermediate filament inclusions disease [22].

Neuroscience researchers are deeply concerned with elucidating the neuronal degeneration and apoptosis associated with human neurological diseases. Accordingly, the neurological mutant *dt/dt *mouse can be adopted to examine the genetic and neurological basis of human diseases, such as peripheral nerve degeneration. The combination of impaired nociception and autonomic dysfunction, in which motor neurons were relatively or completely spared, is characteristic of autosomal recessive autonomic neuropathy [23]. An investigation of changes in peripheral innervation and neuronal number within the autonomic ganglia of *dt/dt *may clarify the pathophysiology of mutation.

In this study, immunohistochemical analyses of cutaneous and iridial tissues, as well as autonomic neuronal counting within ganglia were performed on *dt/dt *mice *in vivo*. Furthermore, to study patterns of neuronal IFs in autonomic neurons of *dt/dt*, sympathetic neurons were collected and assayed *in vitro*. Distribution patterns of neuronal IFs in cultured sympathetic ganglia neurons were studied thoroughly using immunocytochemistry and conventional electron microscopy.

## Materials and methods

### Mice

B6C3Fe*-ala-Dst^dt-J ^*mice, carrying a natural mutation in the *BPAG1 *gene, were utilized in this study. Experimental mice were collected from litters of heterozygous breeding pairs, provided by Jackson Laboratories (Bar Harbor, MA). Care and treatment of animals were in accordance with standard laboratory animal protocols approved by the Animal Care Committee (Chung Shan Medical University). A total of 26 adult mice (10 *dt/dt *and 16 wild-type) were selected by reverse transcriptase-polymerase chain reaction (RT-PCR) assays from litters of nine heterozygous breeding pairs for the following studies.

### RT-PCR assays

Mice were sacrificed by cervical dislocation after anesthesia with choral hydrate (400 mg/kg of body weight, intraperitoneally). Total RNA from the tissue samples was prepared using TRIzol reagent and converted to cDNA using a reverse primer and reverse transcriptase (Invitrogen Corp., Carlsbad, CA). To amplify the cDNA, this study used Taq DNA polymerase and PCR, consisting of 40 cycles at 94°C for 30 sec, 65°C for 30 sec and, 72°C for 1 minute. Specific PCR primer sequences were prepared as follows: *BPAG1n *primers (5'-GAC GAG AAG TCG GTG ATA ACC TAT G-3' and 3'-CTG TTT GAG TAG GAC GGG CTT-5', producing a 511-bp fragment). The primers of *β-actin *applied as the positive control, were 5'-AAC CAT GAG GGA AAT CGY GCA C-3' and 3'-AGT CAA GGG AAT CGG CAG AAT G-5' (producing a 219 bp fragment).

### Immunohistochemistry for nerve tissues in footpads

The eight-week-old mice were anesthetized and perfused with 4% paraformaldehyde. Tissue samples were collected and then cut on a freezing microtome. Floating sections were transferred into phosphate-buffered saline (PBS) solution, incubated in 3.5% hydrogen peroxide to eliminate endogenous peroxidase activity, and finally blocked using 5% normal goat serum and 0.5% Triton X-100 in PBS. Sections were incubated with the primary antibody against neuronal marker proteins such as gene product 9.5 (PGP 9.5, 1: 500, Chemicon, Temecula, CA) at 4°C for 16-24 hours. After rinsing in PBS, sections were incubated with biotinylated secondary antibody of the appropriate specie (Sigma-Aldrich, St. Louis, MO). The color reaction product was accomplished with a Vector ABC kit and with the 3, 3-diaminobenzadine (DAB) reaction (Vector Labs, Burlingame, CA).

### Immunohistochemistry of nerve fibers in iris

To prevent the DAB color reaction from being covered up by pigment granules in the iris, the fluorescence immunohistochemistry was applied. Iridial wholemounts were labeled with pan neuronal marker using fluorescence-labeled secondary antibody. Irises were incubated for 24 hours in the pan neuronal marker primary antibody (PGP 9.5, Chemicon) at 4°C. After washing, tissues were then reacted for 2 hours with FITC-conjugated goat anti-rabbit IgG (Sigma-Aldrich). Flat mounts were analyzed under a Zeiss Axiophot microscope (Carl Zeiss, Oberkochen, Germany).

### Quantifying neuronal number

To perform quantitative analysis of the number of sympathetic neurons, lumbar ganglia were fully sectioned at a thickness of 8 μm. Every tenth section was subjected to examination to avoid double counting of cells, and a total of 15-20 sections were selected for each ganglion. Total number of neurons with both nucleus and nucleolus in the focal plane was counted. Statistical difference was determined by an analysis of Student's *t*-test.

In ciliary ganglia, a different approach was adopted given its small size. Serial sections (8 μm) were stained with hematoxylin, and all neurons were counted throughout every section, covering the entire ciliary ganglia. Only cells with distinct nuclei were counted to avoid double counting of cells.

### Histograms of relative proportions of neuronal areas

For histograms of relative proportions of neuronal areas, the method was modified from the study of dorsal root ganglia [24]. In sympathetic ganglia, the largest cross sections were chosen for cell counting to avoid double counting of cells. In ciliary ganglia, neurons were counted through sections (8 μm) of whole ganglia. The area of neuron with distinct nucleoli was determined. The area of each neuron was determined using the image analysis software (Image-Pro Plus v. 4.5, Media Cybernetics, Silver Spring, MD). For construction of histogram, total counting number of neurons analyzed in each mouse was taken as 100%. Neuronal size was sorted into groups at 50 μm^2 ^intervals and the percentage of neurons falling into these size ranges was calculated.

### Pupillary light reflex

Pupillary responses were measured in unanesthetized age-matched eight-week-old wild-type and *dt/dt *mice. Each animal was allowed to adapt to darkness for at least 30 minutes. Subsequently, mice were placed on a custom-built stereotactic apparatus, by which animal movement was restricted by a 28 mm diameter polyethylene tube. A beam of light was directed to the eye for evaluation of the pupillary light reflex. The pupillary diameter was measured and used to calculate pupil area.

### Cell culture for embryonic neurons from sympathetic ganglia in wild-type and *dt/dt *mice

To determine the effect of neuronal IF on developing sympathetic neurons, sympathetic ganglia were dissected and collected from mouse embryos at embryonic day 15.5. To determine the genotype each embryo from the heterozygous breeding, the spinal cord of each embryo was collected for RT-PCR analysis, as in our previous study [6]. Sympathetic ganglia collected from each embryo were treated with 0.25% trypsin without EDTA for 20 minutes at 37°C. Cells from sympathetic ganglia were physically dissociated by pipetting, plated in culture dishes (Corning, New York, NY), and allowed to attach to coverslips plated with poly-D-lysine (Sigma-Aldrich). The culture medium was composed of Neurolbasal medium (Gibco, Grand Island, NY) supplemented with 20% fetal bovine serum, 2% glucose, 2.5 mM L-glutamine, 2% B-27, and 100 ng/mL nerve growth factor (R & D Systems, Minneapolis, MN). Cultured sympathetic ganglia cells were collected at 5 days *in vitro *(DIV) for further analysis.

### Electron microscopy for cultured neurons

Cultured cells were fixed with a fixative containing 4% paraformaldehyde and 1% glutaraldehyde in 0.1 M cacodylate buffer (pH 7.4). Following post-fixation in 1% osmium tetroxide for 2 hours, tissues were dehydrated through a graded series of ethanol, and then embedded in Epon 812 resin. Ultrathin sections (70 nm-thick) were collected on copper grids, doubly stained with uranyl acetate and lead citrate, and observed under a Hitachi 7100 electron microscope (Hitachi, Tokyo, Japan).

### Immunocytochemistry for cultured neurons from sympathetic ganglia

Embryonic neurons were cultured on poly-D-lysine coated glass coverslips in a cell culture dish. Cultured neurons were fixed in methanol for 30 minutes at 4°C and then permeabilized with 0.1% Triton X-100 in PBS for 5 minutes. After which, cells were incubated for 1 hour with primary antibodies against ubiquitin and medium-neurofilament (NF-M; Sigma-Aldrich), followed by washing three times in PBS. Samples were then incubated with secondary antibodies and Hoechst 33342 (Sigma-Aldrich) at 27°C for 1 hour. Hoechst 33342 was applied to stain nuclei. Subsequently, cultured neurons were mounted and examined under a Zeiss LSM 510 META confocal spectral microscope (Oberkochen, Germany).

## Results

### Genetic characterization of *dt/dt *mice

**Figure 1:**
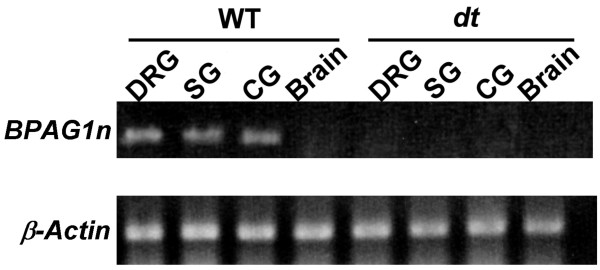
**RT-PCR analysis of *BPAG1n *and *β-actin *mRNAs from wild-type and *dt/dt *mice**. *BPAG1n *mRNA could be detected in dorsal root ganglia, sympathetic ganglia, and ciliary ganglia of wild-type mice, but not in *dt/dt *mice. β-Actin primers were used as positive controls.

This study initially determined the expression patterns of *BPAG1n *mRNA from wild-type and *dt/dt *mice by RT-PCR. The *BPAG1n *mRNA could be detected in the dorsal root, sympathetic, and ciliary ganglia of wild-type mice, but not in that of *dt/dt *mice (Figure 1).

### Sympathetic denervation in the sweat gland of *dt/dt *mice

**Figure 2:**
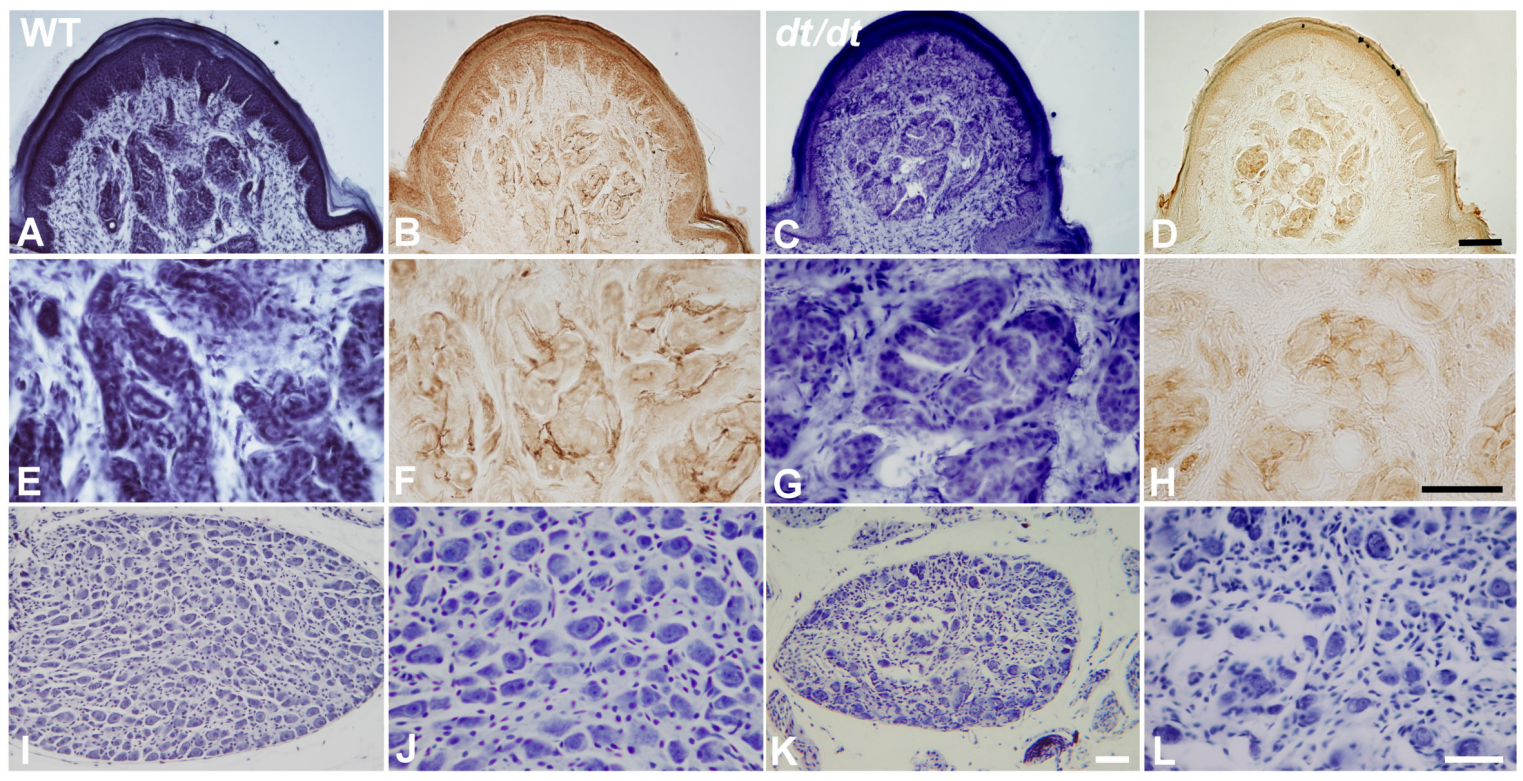
**Localization of sympathetic nerves around sweat glands and neurons in sympathetic ganglion of wild-type and *dt/dt *mice**. Serial sections of footpads were stained with hematoxylin or antibody against PGP 9.5 in wild-type (A, B, E and F) and *dt/dt *mice (C, D, G and H). In normal mice skin, numerous PGP 9.5-immunoreactive autonomic fibers were visible in dermis (A and B). Conversely, only some nerve fibers were identifiable in *dt/dt *mice (C and D). High-power photomicrographs revealed that autonomic nerves innervated sweat gland and displayed dense and strong PGP 9.5 immunoreactivity in normal skin (E and F), whereas only fragmented autonomic nerves could be observed in *dt/dt *mice (G and H). From the observation of lumbar sympathetic ganglia, many neurons could be recognizable in the section of ganglia of wild-type mice (I and J). However, only fewer neurons could be found in *dt/dt *mice compared with wild-type mice (K and L). Scale bars = 40 μm in A-H; 50 μm in I-L.

To investigate sympathetic innervation, the skin of the footpad was immunoassayed using the antibody against PGP 9.5. In wild-type mice, various immunopositive nerves encircled the coiled tubules of sweat glands, forming an interlacing, dark, and continuous pattern (Figure 2A and 2B). In *dt/dt *mice, a few faintly stained immunopositive nerves were identified in the dermis of footpads (Figure 2C and 2D). In normal mice, numerous autonomic nerves encircled innervated sweat glands (Figure 2E and 2F). However, sweat glands were significantly denervated, with only weak and disorganized immunoreactivity around them (Figure 2G and 2H). This observation, it may be implied that autonomic nerves innervated sweat glands were poor in *dt/dt*. Histopathological analysis revealed that sweat glands in *dt/dt *mutants were not significantly changed. The morphology of sweat glands in *dt/dt *mutants does not differ in appearance compared with that in wild-type mice (Figure 2E and 2G).

Additionally, the morphology of lumbar sympathetic ganglia was investigated. Typical sympathetic neurons with visible nucleoli were observed in wild-type mice (Figure Figures. 2I and 2J). The neuronal number was significantly reduced upon observation under quantitative analysis (Table 1 and Figure 2K), and more glial cells could be easily identified in the ganglia of *dt/dt *mice (Figure 2L).

### Density of parasympathetic nerve significantly decrease in the iris of *dt/dt *mice

**Figure 3:**
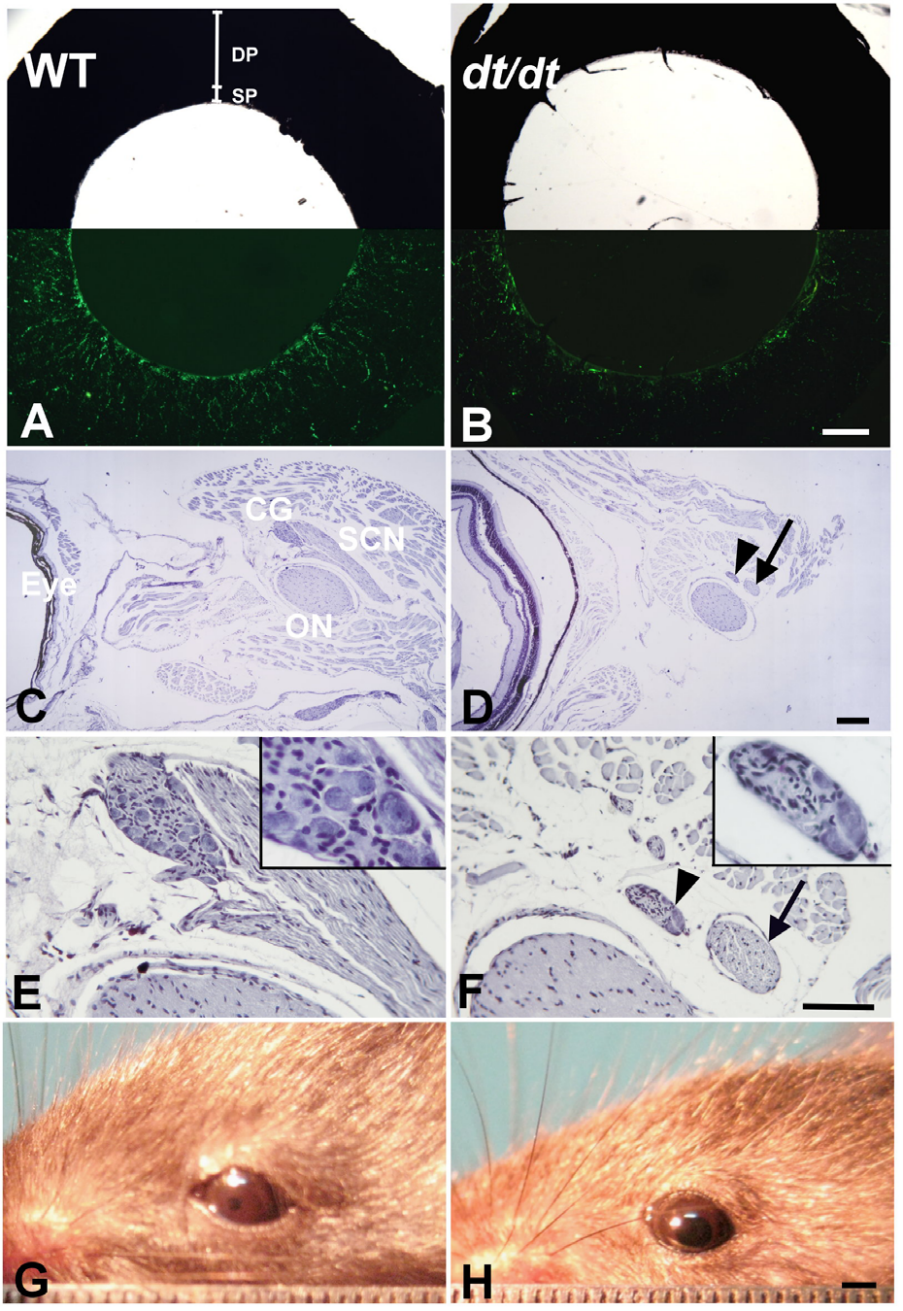
**Nerve degeneration in irises and notably wider pupils in response to light of *dt/dt *mice**. Wholemount preparations of irises were stained by immunofluorescence for pan neuronal marker PGP 9.5 (A and B). In wild-type mice, PGP 9.5-positive fibers were circumferentially distributed along the pupillary ruff in the sphincter pupillae (SP) area and were radially oriented toward the pupil in the dilator pupillae (DP) area (A). Compared with intact wild-type mice, a few remaining immunopositive fibers exhibited marked decrease in density throughout the sphincter and dilator area in *dt/dt *mice (B). Ciliary ganglion (CG) and short ciliary nerve (SCN) could be found along the outer surface of the optic nerve (ON) in wild-type mice (C). However, the smaller nerve bundle (arrow) and ganglion (arrowhead) could be observed in *dt/dt *mice (D). High-power photomicrographs revealed that the ganglion with typical neuronal morphology was observed in wild-type mice (E), whereas the ganglion with fewer number and smaller size of neurons could be found in *dt/dt *mice (F). To investigate the denervation effect in the iris of *dt/dt *mice, the light-induced pupillary reflex was tested. The pupillary diameter was narrower in wild-type mice during the pupillary reflex test (G), whereas the pupil was notably wider and iris constriction was weaker in response to light in *dt/dt *mice (H). Scale bars = 200 μm in A-F; 2 mm in G and H.

In irises, the wider diameter of pupil size was noticeable in *dt/dt *mice (Figure 3A and 3B). Dual autonomic innervation occurred in both sphincter and dilator muscles of the iris. In the wholemount iris of *dt/dt *mice, immunopositive fibers showed a marked decrease in density throughout the sphincter and dilator area compared with the intact control iris from wild-type mice (Figure 3A and 3B).

Parasympathetic ciliary ganglion and short ciliary nerve running along the outer surface of the optic nerve could be identified in wild-type mice (Figure 3C). In contrast, the smaller ciliary ganglion and ciliary nerve bundle could be found in *dt/dt *mice (Figure 3D). To illustrate the relationship between the denervation and parasympathetic neuropathy of *dt/dt *mice, neurons in ciliary ganglia were examined as well. We found that the neuronal number was reduced in ciliary ganglia of *dt/dt *mice (Table 1, Figure 3E and 3F). These observations suggest that the parasympathetic innervation of irises is poorer in *dt/dt *mice compared with those in wild-type mice. To investigate the functional defect of autonomic denervation in irises of *dt/dt *mice, the light-induced pupillary reflex was examined. From the pupillary reflex function test, the pupillary diameter size was notably wider and the iris constriction was weaker in terms of the response to light in *dt/dt *mice compared with that in wild-type mice (Figure 3G and 3H).

**Table 1:** Number of neurons in young adult *dt/dt *mice compared with those in age-matched wild-type mice

	Region	Neuronal number	
		wild-type	*dt/dt*
Types of neuron	Lumbar sympathetic ganglia	2147 ± 131	736 ± 362*
	Ciliary ganglia	187 ± 9	80 ± 29*

The neuronal numbers of ganglia were calculated from wild-type (n = 5) and *dt/dt *(n = 4) mice. Neurons with both the nucleus and nucleolus in the focal plane were counted. Results are expressed as mean ± SD and *indicates a value statistically different (*t*-test, P < 0.01) from the wild-type control. The neuronal number in sympathetic ganglia and parasympathetic ciliary ganglia of *dt/dt *is significantly reduced compared with those of wild-type mice.

### Decrease in neuron size in sympathetic ganglia and ciliary ganglia of *dt/dt *mice

**Figure 4:**
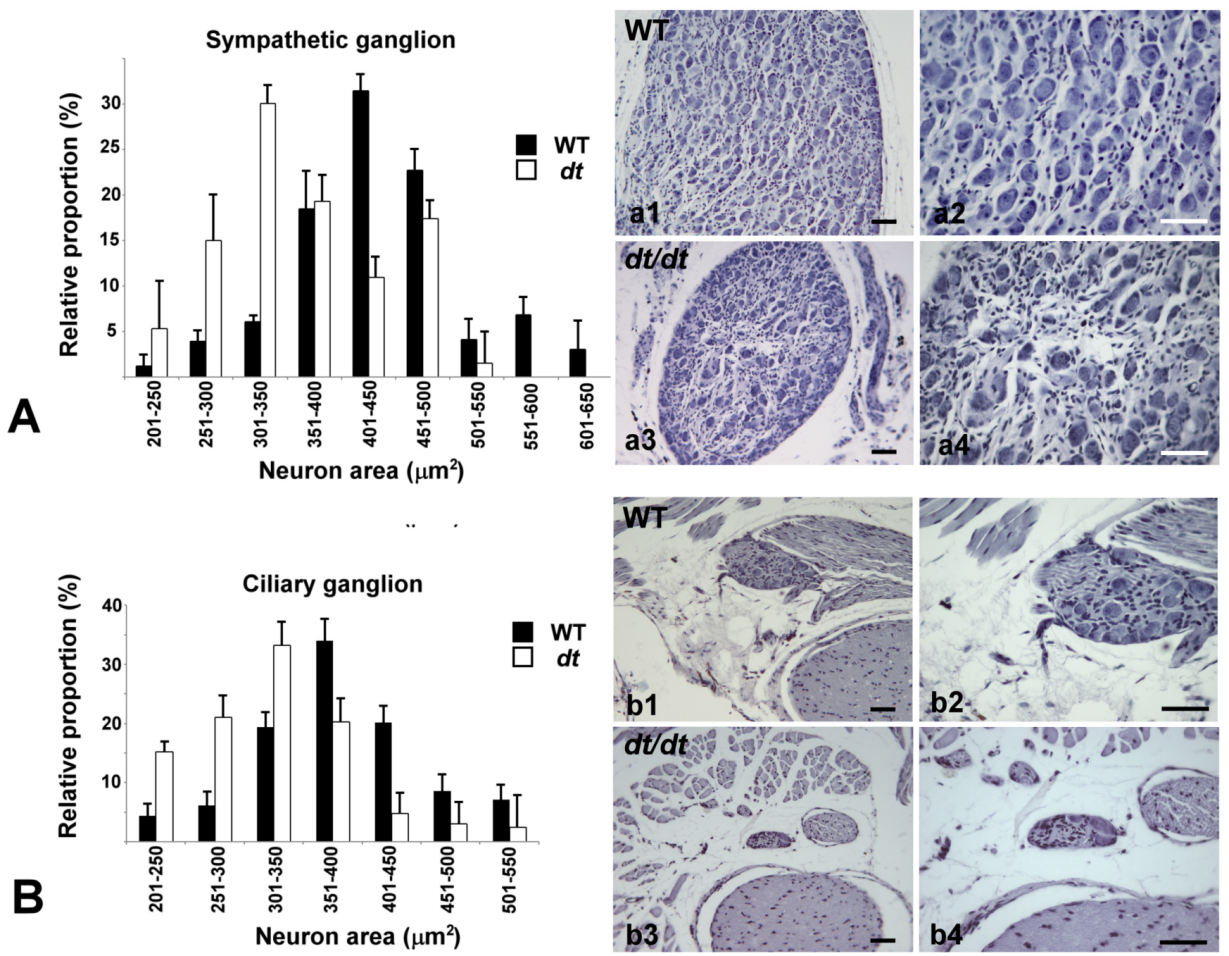
**Histograms of relative proportions of neuron area in wild-type and *dt/dt *mice**. Neuron areas in sympathetic ganglia and in ciliary ganglia of wild-type (n = 5) and *dt/dt *(n = 4) mice were determined (A and B). Neuron areas were sorted into groups at 50 μm^2 ^intervals and the percentage distributions of neuron sizes were divided into classes of the same size range. The greatest proportion of sympathetic neuron ranged between 401 and 450 μm^2 ^in wild-type mice, whereas the greatest proportion ranged between 301 and 350 μm^2 ^in *dt/dt *mice (A). Photomicrographs revealed that the reduced size of sympathetic ganglion and neuron in *dt/dt *mice compared with those in wild-type mice (a1-a4). Moreover, the greatest proportion of neuron ranged between 351 and 400 μm^2 ^in ciliary ganglia of wild-type mice, whereas the greatest proportion ranged between 301 and 350 μm^2 ^in *dt/dt *mice (B). Photomicrographs reappeared that the smaller size of ciliary ganglia and neurons in *dt/dt *mice compared with those in wild-type mice (b1-b4). Scale bars = 100 μm.

To examine the difference in neuronal size of autonomic ganglia between *dt/dt *and wild-type mice, we quantified the cross-sectional areas of neurons in sympathetic ganglia and in ciliary ganglia of *dt/dt *and wild-type mice. Histograms of relative proportions documented a large peak between 401 and 450 μm^2 ^in wild-type mice, whereas between 301 and 350 μm^2 ^in *dt/dt *mice (Figure 4A and 4a1-a4). The greatest proportion of neuron area in ciliary ganglia ranged between 351 and 400 μm^2 ^in wild-type mice, whereas the greatest proportion ranged between 301 and 350 μm^2 ^in *dt/dt *mice (Figure 4B and 4b1-b4). Besides neuronal loss of both sympathetic and ciliary ganglia, our data also revealed a decrease in neuron size in sympathetic and ciliary ganglia of *dt/dt *mice.

### Neuronal IF aggregates and apoptosis-like death of cultured sympathetic neurons from *dt/dt *embryos

**Figure 5:**
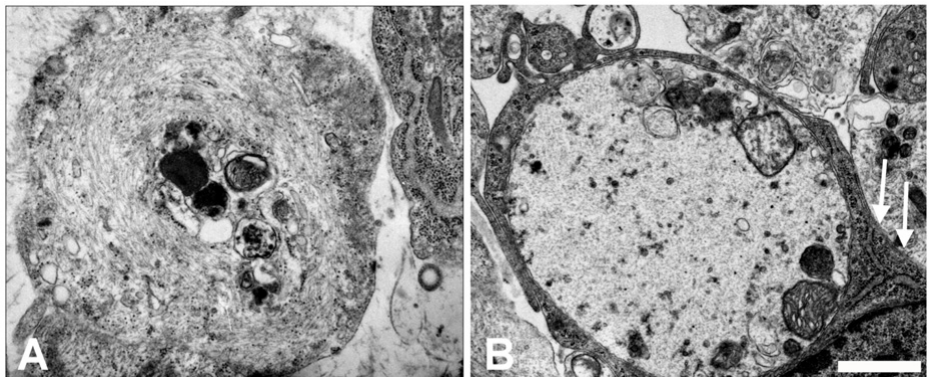
**Ultrastructural patterns of neuronal IFs aggregates in degenerating cultured sympathetic neurons from *dt/dt *embryos**. At the ultrastructural level, IF aggregates and randomly oriented IFs were observed in cultured sympathetic neurons from embryonic *dt/dt *mice (A). Neuronal IFs formed aggregates in soma, suggesting its involvement in the degeneration of neurons from *dt/dt *mice. Random orientation of IFs and axonal organelles was observed in the swelling processes of sympathetic neurons from *dt/dt *embryos (B). The swelling process was surrounded by a Schwann cell (arrows, B). Scale bars = 1 μm.

In cultured sympathetic neurons from *dt/dt *embryos at 5 DIV, massive accumulation of neuronal IFs could be observed in cell processes (Figure 5A and 5B). The density of IFs was very high and the pattern of IFs was randomly oriented. Some entrapped organelles together with IF aggregates were found in the cellular process of cultured sympathetic neurons from *dt/dt *mutants (Figure 5A).

**Figure 6:**
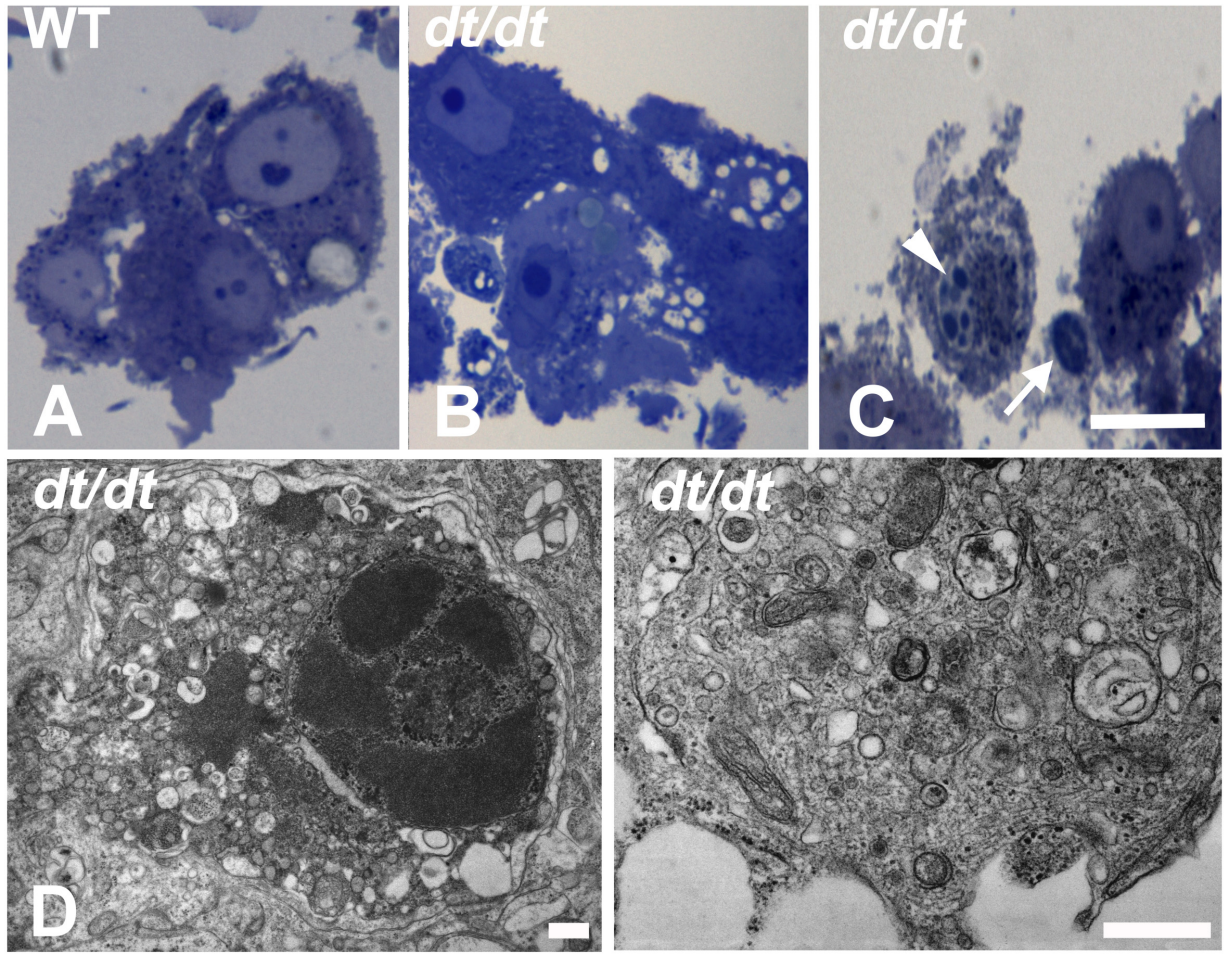
**Vacuolization and chromatin condensation of cultured sympathetic neurons from *dt/dt *mice**. In semithin sections, the morphological patterns of cultured neurons were normal from wild-type mice (A). However, certain membrane-bounded vesicles in the perikaryon (B) and chromatin condensation (arrowhead, C) could be found in cultured neurons of *dt/dt *mutants. Aside from sympathetic neurons, a Schwann cell could be also found in this primary culture (arrow, C). At the ultrastructural level, images of cultured neurons reveal autophagic structures and prominent vacuolization from *dt/dt *mice (D and E). Furthermore, the apoptosis-like characteristic of chromatin condensation with intact nuclear envelope and cell membrane could be observed from *dt/dt *embryos (D). Multi-membraned autophagosomes could be found in the cytoplasm of *dt/dt *mutants (E). Scale bars = 20 μm in A-C; 1 μm in D and E.

Morphological patterns of cultured sympathetic neurons from wild-type mice were normal (Figure 6A). However, prominent vacuolization, typical autophagosomal structures and condensed chromatin could be found in cultured neurons of *dt/dt *mice under light and electron microscopy (Figure 6B-E). Multi-membraned structures, including late lysosomes and autophagosomes, could be found in the cultured neurons, suggesting that cells are attempting to clean up the damaged organelles (Figure 6E). Some cultured neurons with numerous vacuolizations in cytoplasma of *dt/dt *exhibited apoptosis-like death (Figure 6D). The chromatin condensation with intact cell membrane could be observed in degenerative neurons from *dt/dt *(Figure 6C and 6D).

### Patterns of ubiquitin in degenerating neuron with IFs accumulation

**Figure 7:**
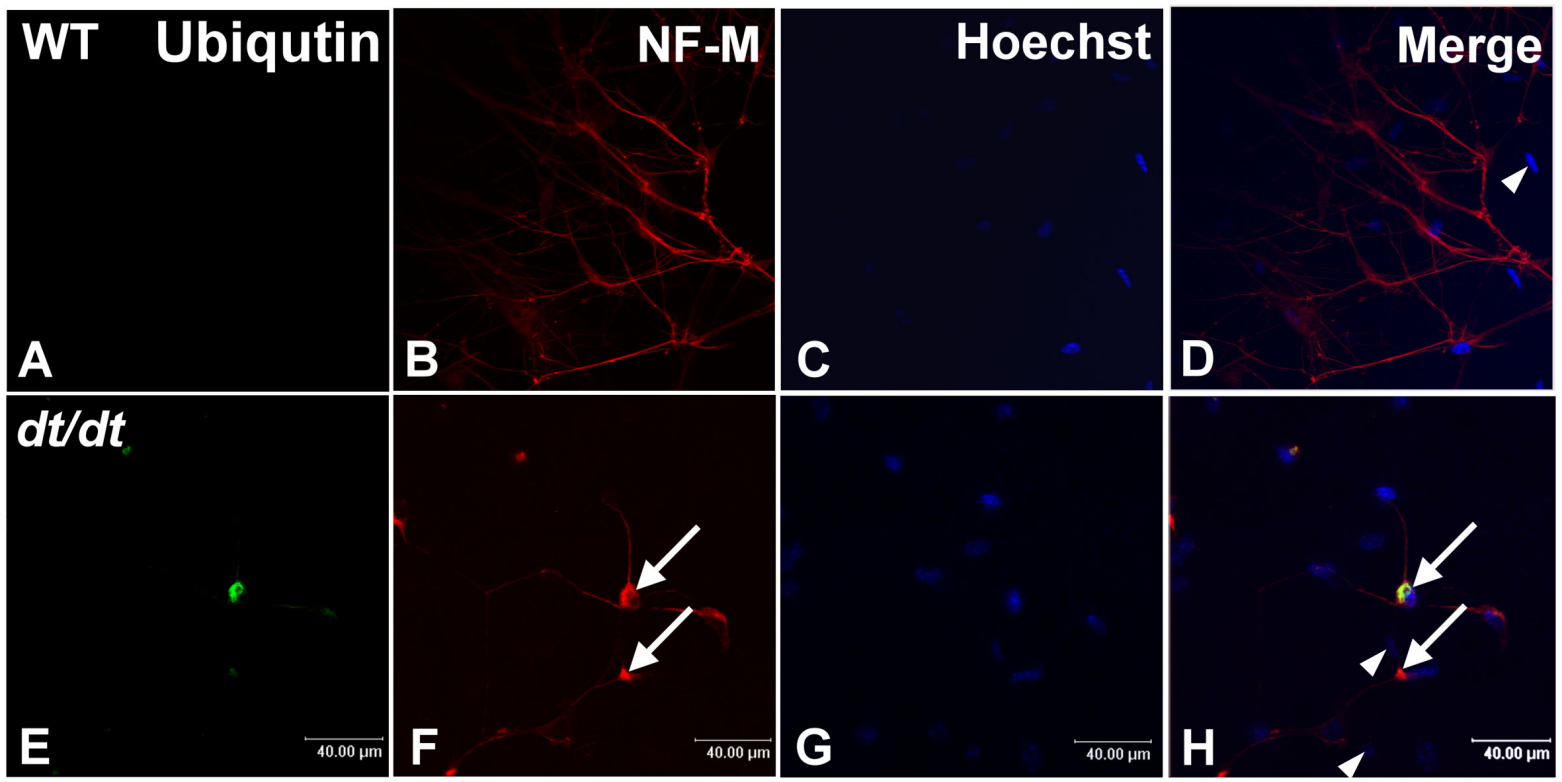
**Immunoreactivity of ubiquitin and NF-M in cultured neurons from wild-type and *dt/dt *mice**. Cultured neurons were double-labeled with antibodies against ubiquitin (green) and neuronal intermediate filament protein NF-M (red), and their nuclei were stained with Hoechst 33342 (blue). The ubiquitin-positive reaction was hardly noticeable in neurons of wild-type mice (A-D). Cultured neurons with abnormal accumulations of NF-M were mostly observed in the proximal region of axons and within cell bodies of cultured sympathetic neurons from *dt/dt *mutants (F, arrows). Some neurons with NF-M accumulations could also be labeled with the antibody against the ubiquitin (E-H). Some smaller nuclei of non-neuronal cells were also observed in the primary culture (arrowheads, D and H). Scale bars = 40 μm.

To determine the relationship between IFs and degrading proteins, NF-M and ubiquitin were examined by immunocytochemistry. At 5 DIV, cultured sympathetic neurons from wild-type mice highly expressed NF-M, but not ubiquitin (Figure 7A-D). Neuronal intermediate filament protein NF-M was normally distributed in axonal processes. However, the two proteins of ubiquitin and NF-M could be colocalized in the perikaryon of cultured sympathetic neurons from *dt/dt *mice (Figure 7E-H). Based on confocal microscopy, the distribution of ubiquitin protein was associated with the abnormal accumulation of neuronal IFs aggregates in degenerative sympathetic neurons from *dt/dt *mutants.

## Discussion

### Autonomic denervation in sweat glands and irises of *dt/dt *mice

Previous studies revealed the expression of *BPAG1n *in a variety of sensory and motor neurons from the embryonic to the postnatal stage in normal development. However, morphometric study has shown sensory innervations is significantly reduced in *dt/dt *mutants [3,5,7,8]. This study indicates that the sensory nerve is not only markedly denervated in the cutaneous part of footpads, but that sympathetic innervation is also severely impaired in sweat glands of young adult *dt/dt *mice. The sympathetically innervated sweat glands substantially degenerated in footpads of *dt/dt *mice. This degeneration pattern was demonstrated with immunohistochemistry using general neuronal marker PGP 9.5. Our new finding of the sympathetic denervation adds another criterion for phenotyping *dt/dt *mice.

Ciliary ganglion, like sympathetic ganglion, is a neural crest-derived parasympathetic ganglion [25,26]. From our observation, the neuronal number of ciliary ganglion was significantly decreased in *dt/dt *mice. Moreover, the functional assay provides compelling evidence regarding denervation of irises and the wider iridial diameter of pupillary response to light in *dt/dt *mice. Based on these findings, we hypothesize that *BPAG1 *gene has an important role in the normal development of the ciliary ganglion. The loss of BPAG1n, a cytoskeleton linker protein, in neurons of sympathetic and parasympathetic ganglia suggests that the cytoskeletal dysfunction may trigger the neuronal death during cell migration. This phenomenon may account for the expression of *BPAG1n *in numerous neurons during normal development, but neuronal degeneration is limited to peripheral neurons derived from neural crest cells in *BPAG1*-deficient mice.

The autonomic system is considered unaffected by neurodegenerative disorders such as X-linked recessive spinobulbar muscular atrophy and Guillain-Barre syndrome, but observations have revealed autonomic skin denervation [27,28]. This investigation also demonstrated the sympathetic denervation of sweat glands in footpads and parasympathetic denervation of irises in eyes of *dt/dt *mutants. The terminal endings of the sympathetic nerve commonly degenerate more quickly than the proximal portions of the degenerating sympathetic ganglia neurons [29]. Skin denervation studies have established an early sign of neuropathy before ganglionopathy is detected [30]. From our studies, cutaneous tissues and iridial wholemounts with immunohistochemical analysis constitute a reliable approach for distinguishing between neuropathy and neuronopathy. Our data provides an evidence of epidermal and iridial denervation in footpads and eyes with autonomic neuropathy in neuronal cytoskeletal dysfunction.

### Roles of neuronal cytoskeletons in cultured sympathetic neurons from *dt/dt *embryos

Clinical and basic neuropathy has indicated that neurodegenerative disorders are morphologically represented by progressive neuronal damage and are associated with the typical cytoskeleton dysfunction [15,16,20,21]. Other results have also indicated that abnormal aggregations of IF proteins are significantly involved in the mechanism of neuronal death [22,31,32]. In the previous study of *dt/dt *mice, the abnormal accumulation of IFs in degenerating primary sensory neurons was observed *in vivo *and *in vitro *[7]. The abnormal accumulation of neuronal IF proteins may impair axonal transport and later trigger neuronal apoptosis cascade of neurons in dorsal root ganglia of *dt/dt *[7]. In our current study, abnormal translocation of neuronal IFs was also found in the nerve process and soma of cultured sympathetic neurons from *dt/dt *embryos. It suggests that the deficiency in BPAG1, the cytoskeletal linker protein, may induce neuronal death in the sympathetic nervous system of *dt/dt *mice during development.

### Protein degradation in degenerating neurons from *dt/dt *mutants

Intracellular protein degradation is mainly mediated by the ubiquitin-proteasome and autophagy-lysosome systems in eukaryotic cells [33,34]. Ubiquitin-proteasome system is chiefly responsible for degrading short-lived proteins and a selective form of catabolism [33]. Repetition of the cycle generates polyubiquitin chains on target proteins, which are then degraded into smaller peptides. In contrast, autophagy is a broad term for the degradation of long-lived proteins and a nonselective form of catabolism [34]. Some studies have revealed that abnormal protein aggregations, which are potential toxins, could be quickly degraded by the ubiquitin-proteasome and autophagy-lysosome systems [35,36]. Our immunomicroscopy images show the involvement of ubiquitin in degenerating neurons from *dt/dt*. In addition, preliminary transmission electron micrographs reveal lysosomal or autophagosomal structures and pronounced vacuolization in the cultured sympathetic neurons. Based on our observation, both ubiquitin-proteasome and autophagy-lysosome systemsmayhave essential roles in degrading neuronal IFs aggregations in sympathetic neurons of *dt/dt *mutants.

## Conclusion

We have demonstrated the epidermal and iridial denervation associated with autonomic neuropathy of *dt/dt *mutants. Additionally, abnormally aggregated neuronal IFs may participate in neuronal death of cultured autonomic neurons from *dt/dt *mutants. Our results suggest that a deficiency in the cytoskeletal linker BPAG1 is responsible for dominant sensory nerve degeneration and severe autonomic degeneration in *dt/dt *mice.

## Abbreviations

dt: dystonia musculorum; BPAG1: bullous pemphigoid antigen 1; BPAG1n: neural isoform of BPAG1; PGP 9.5: protein gene product 9.5; IFs: intermediate filaments; RT-PCR: reverse transcriptase-polymerase chain reaction; PBS: phosphate-buffered saline; DAB: 3, 3-diaminobenzadine; NF-M: medium-neurofilament;

## Competing interests

The authors declare that they have no competing interests.

## Authors' contributions

KWT and CLC designed, carried out the main experiment and drafted the manuscript. MLP helped design the experiment and improve the manuscript. YJW and KJL participated in immunohistochemistry assay and statistical analysis. All authors read and approved the final manuscript.
